# Obesity and Diabetes as Risk Factors for Severe *Plasmodium falciparum* Malaria: Results From a Swedish Nationwide Study

**DOI:** 10.1093/cid/cix437

**Published:** 2017-05-16

**Authors:** Katja Wyss, Andreas Wångdahl, Maria Vesterlund, Ulf Hammar, Saduddin Dashti, Pontus Naucler, Anna Färnert

**Affiliations:** 1 Unit of Infectious Diseases, Department of Medicine Solna, Karolinska Institutet, and Departments of; 2 Emergency Medicine and; 3 Infectious Diseases, Karolinska University Hospital, Stockholm,; 4 Department of Infectious Diseases, Västerås Central Hospital, and; 5 Unit of Biostatistics, Department of Epidemiology, Institute for Environmental Medicine, Karolinska Institutet, Stockholm, Sweden

**Keywords:** severe malaria, comorbidity, noncommunicable diseases, diabetes, obesity

## Abstract

**Background:**

Noncommunicable diseases and obesity are increasing in prevalence globally, also in populations at risk of malaria. We sought to investigate if comorbidity, in terms of chronic diseases and obesity, is associated with severe *Plasmodium falciparum* malaria.

**Methods:**

We performed a retrospective observational study in adults (≥18 years of age) diagnosed with malaria in Sweden between January 1995 and May 2015. We identified cases through the surveillance database at the Public Health Agency of Sweden and reviewed clinical data from 18 hospitals. Multivariable logistic regression was used to assess associations between comorbidities and severe malaria.

**Results:**

Among 937 adults (median age, 37 years; 66.5% were male), patients with severe malaria had higher prevalence of chronic diseases (28/92 [30.4%]) compared with nonsevere cases (151/845 [17.9%]) (*P* = .004). Charlson comorbidity score ≥1 was associated with severe malaria (adjusted odds ratio [aOR], 2.63 [95% confidence interval {CI}, 1.45–4.77), as was diabetes among individual diagnoses (aOR, 2.98 [95% CI, 1.25–7.09]). Median body mass index was higher among severe (29.3 kg/m^2^) than nonsevere cases (24.7 kg/m^2^) (*P* < .001). Obesity was strongly associated with severe malaria, both independently (aOR, 5.58 [95% CI, 2.03–15.36]) and in combination with an additional metabolic risk factor (hypertension, dyslipidemia, or diabetes) (aOR, 6.54 [95% CI, 1.87–22.88]). The associations were observed among nonimmune travelers as well as immigrants from endemic areas.

**Conclusions:**

Comorbidities, specifically obesity and diabetes, are previously unidentified risk factors for severe malaria in adults diagnosed with *P. falciparum*. Noncommunicable diseases should be considered in the acute management and prevention of malaria.

## Summary

In this nationwide observational study of 937 adults diagnosed with *Plasmodium falciparum* malaria in Sweden, Charlson comorbidity score ≥1 as well as diabetes and obesity were significantly associated with severe malaria in both nonimmune travelers and immigrants from endemic countries.

Obesity and noncommunicable diseases (NCDs), such as diabetes, hypertension, and cardiovascular disease, have increased globally, including in malaria-endemic regions [1]. In addition, a significant proportion of travelers are older [2], and an estimated one-third of travelers to malarious countries have underlying medical conditions [3]. This changing disease panorama in populations at risk of malaria warrants the need to establish how comorbidities affect severity of malaria.

Studies on comorbidities have largely focused on coinfections, with human immunodeficiency virus (HIV) and chronic hepatitis B reported to influence the risk of severe malaria [4, 5]. The role of NCDs has only been assessed in a few studies. Overweight was observed to affect disease course in uncomplicated cases in Thailand [6], and higher prevalence of asymptomatic *Plasmodium falciparum* infections was found among individuals with type 2 diabetes in Ghana [7].

Old age has been identified as a risk factor for both severe and fatal malaria in travelers [8–11], and longer hospital stay was reported in patients aged ≥65 years with chronic diseases [12]. However, no study has systematically assessed how comorbidities affect severity of malaria. Such evaluation is important to improve public health strategies and support clinicians to recognize patients at risk.

In this nationwide observational study of imported malaria in Sweden over 20 years, we assessed if comorbidity, and any chronic condition in particular, is associated with severe malaria in adults diagnosed with *P. falciparum*.

## METHODS

### Study Population

Cases were identified through national surveillance at the Public Health Agency of Sweden. Malaria is a notifiable disease in Sweden with mandatory reporting by diagnosing clinicians and microbiology laboratories, providing high detection sensitivity [13]. All adults (≥18 years of age) with a first episode of microbiologically confirmed *P. falciparum* and complete medical records from 1 January 1995 to 12 April 2013 were included, and for Umeå until 31 August 2013 and Stockholm until 31 May 2015. Patients without symptoms and asexual parasites after treatment elsewhere were excluded.

### Data Collection

Medical records from identified cases were provided by 18 hospitals managing malaria in Sweden. Data were retrieved regarding sociodemographics, travel history, chemoprophylaxis, clinical presentation, comorbidities, patient and healthcare delay (days from symptoms onset until healthcare contact, and from healthcare contact to diagnosis), intensive care, duration of hospital stay, treatment, and outcome, as well as routine blood chemistry and microbiology data including parasitemia, HIV, and hepatitis status. Data on weight and height were collected in Stockholm and Umeå. Medication lists and previous *International Classification of Diseases* (*ICD*) codes in electronic medical records were reviewed to capture additional chronic diseases.

### Malaria Diagnosis

Malaria was diagnosed by microscopy of thick and thin blood films stained with Giemsa or Field stain. Parasite species were occasionally determined by polymerase chain reaction. Parasitemia (percentage of infected erythrocytes) was estimated in thin smears, or by counting parasites in thick films either against leukocytes or ocular fields.

### Primary Outcome

Severe malaria was defined using 2012 World Health Organization (WHO) criteria [14], with minor modifications [15] (Table 1), and hyperparasitemia >5% according to previous WHO definition [16]. A sensitivity analysis without hyperparasitemia as single criterion was also performed.

**Table 1.** Criteria for Severe *Plasmodium falciparum* Malaria According to the World Health Organization Definition^a^ With Modifications^b^

Clinical/Laboratory/ Radiological Finding	Specification
Impaired consciousness or unrousable coma	Glasgow Coma Scale ≤10^b^
Multiple convulsions	>2 episodes within 24 h or generalized seizures
Respiratory distress or acidotic breathing	Requirement of noninvasive or endotracheal mechanical ventilation^b^ or respiratory rate of ≥40 breaths/min on room air
Circulatory collapse or shock	Systolic blood pressure <80 mm Hg or ≤80 mm Hg despite volume repletion
Acute pulmonary edema	Verified radiologically
Acute respiratory distress syndrome	Verified radiologically
Acute kidney injury/renal impairment	Serum creatinine >265 μmol/L
Acidosis or hyperlactatemia	pH <7.25 or plasma bicarbonate <15 mmol/L or lactate >5 mmol/L
Clinical jaundice plus evidence of other vital organ dysfunction	Bilirubin >50 μmol/L together with circulatory instability, respiratory distress, impaired consciousness, severe coagulopathy, or acute kidney injury
Severe normocytic anemia	Hemoglobin <70 g/L not related to other cause than the malaria infection
Abnormal bleeding	Spontaneous bleeding from gums, mouth, or gastrointestinal tract
Macroscopic hemoglobinuria	Unequivocally related to acute malaria and verified by urine dipstick
Hypoglycemia	Blood glucose <2.2 mmol/L
Hyperparasitemia	Parasitemia >5%

^a^Criteria according to World Health Organization (WHO) management of severe malaria 2012 [14], and hyperparasitemia according to 2000 WHO definition [16].

^b^Modifications according to Bruneel et al [15].

### Covariables

Medical conditions were categorized according to the *ICD, Tenth Revision* (*ICD-10*). Comorbidity was assessed both as individual diagnoses and as severity-weighted scores using the Charlson comorbidity index adjusted to *ICD-10* [17], and with HIV without AIDS given a score of 1 [18]. Only chronic diseases present at the time of malaria diagnosis and history of malignancies were included in the analysis. Previous resolved conditions such as pneumonia or appendectomy were not included.

For patients with weight and height recorded at time of malaria diagnosis, body mass index (BMI) was calculated as weight in kilograms divided by square height in meters, and categorized according to WHO’s BMI classification for adults [19]. Obesity was defined as BMI ≥30 (WHO obesity class I–III).

Metabolic syndrome was defined according to the International Diabetes Federation as BMI ≥30 together with 2 additional metabolic risk factors: diabetes, dyslipidemia, and/or hypertension [20].

Individuals born in countries with high malaria transmission in sub-Saharan Africa [21] were referred to as of “endemic origin,” and all others as “non/low endemic origin.” Duration of residency in a malaria-free country for patients of endemic origin was categorized as <15 and ≥15 years, based on previous findings [22].

### Statistical Analysis

Statistical analyses were performed using Stata version 13 software (StataCorp). Categorical data were compared using χ^2^ or Fisher exact test, and continuous data using Wilcoxon-Mann-Whitney test. Univariable and multivariable logistic regression were used to assess if comorbidity was associated with severe malaria. Age and endemic origin were included as possible confounders in all multivariable analyses based on biological plausibility. Additional patient characteristics affecting severity in univariable analysis (with *P* < .20) were included in the multivariable model to further adjust for confounding. Factors not associated with severity (*P* > .05) and not changing the effect measure of the main exposures were subsequently excluded. Age was included as continuous variable, after confirming linearity. Potential interactions were tested between variables in the final model. Maximum likelihood ratio test was used to determine best model fit. To account for missing BMI, a multiple imputation model with chained equations was performed based on variables related to severe malaria, obesity status, and missing BMI.

### Ethical Considerations

The study was approved by the ethical review board in Stockholm, Sweden (2009/1328–31/5, 2010/1080-32, and 2012/1155-32).

## RESULTS

### Patient Characteristics

**Figure 1.**
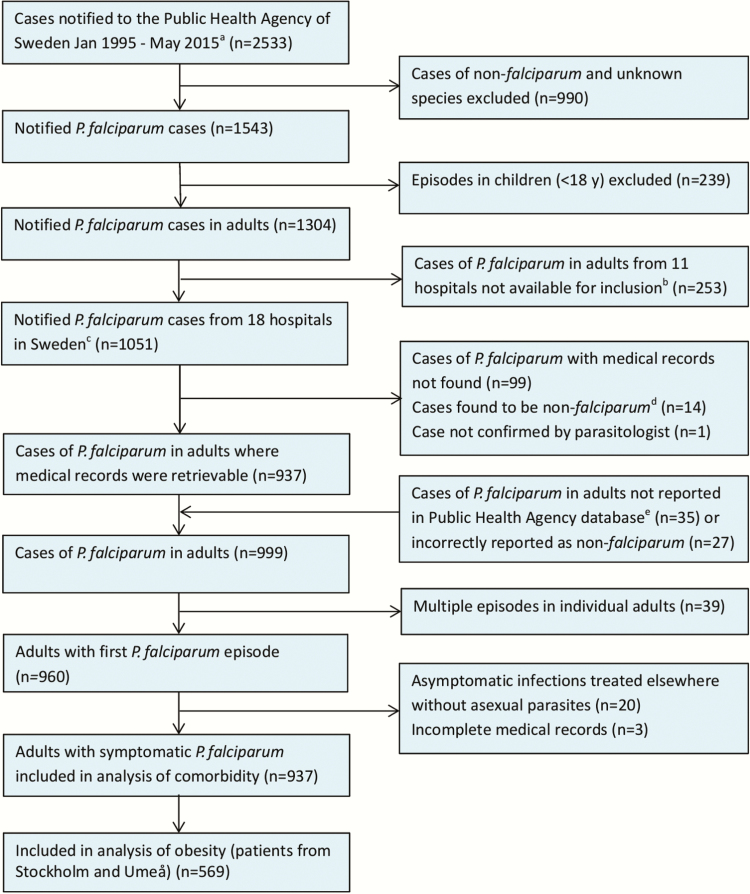
Flowchart of included and excluded patients in the study population. ^a^For Stockholm until May 2015, for Umeå until August 2013; for all other sites until April 2013. ^b^Falun, Helsingborg, Jönköping, Karlstad, Kristianstad, Lund, Malmö, Sunderbyn, Skövde, Trollhättan, Östersund. ^c^Borås, Eskilstuna, Gävle, Göteborg, Halmstad, Kalmar, Karlskrona, Linköping, Norrköping, Skellefteå, Stockholm, Sundsvall, Umeå, Uppsala, Visby, Västerås, Växjö, Örebro. ^d^Cases found to be non-*falciparum* after review of medical records and microbiology results (incorrectly reported as *Plasmodium falciparum* to Public Health Agency of Sweden). ^e^Additional cases with medical records provided by diagnosing hospitals and laboratories that had not been notified to Public Health Agency of Sweden.

In total, 937 adults with *P. falciparum* malaria were included, representing 71.9% (937/1304) of all notified *P. falciparum* cases in adults during the study period (Figure 1). Median age was 37 years (range, 18–83 years), and most were male (66.5%). Five hundred forty-seven patients (58.4%) originated in sub-Saharan Africa; 441 (80.6%) were Swedish residents and 98 (17.9%) newly arrived immigrants or temporary visitors. Among the 388 patients of non/low endemic origin, 342 (88.1%) were from Sweden. Infections were predominantly acquired in Western and Eastern Africa (Table 2).

**Table 2.** Characteristics of Adult Patients with *Plasmodium falciparum* Malaria, According to Disease Severity

Characteristic	No. (%) of Patients			*P* Value^b^	OR (95% CI)
	Total (N = 937)	Nonsevere Malaria (n = 845)	Severe Malaria^a^ (n = 92)		Unadjusted^c^
Age, y					
Median (range)	37 (18–83.3)	36.8 (18–83.3)	44.4 (19.4–75)	<.001	
18–29	241 (25.7)	231 (27.3)	10 (10.9)	<.001	1 (ref)
30–39	302 (32.2)	281 (33.3)	21 (22.8)		1.73 (.80–3.74)
40–49	209 (22.3)	183 (21.7)	26 (28.3)		3.28 (1.54–6.98)
50–59	133 (14.2)	112 (13.3)	21 (22.8)		4.33 (1.97–9.51)
≥60	52 (5.6)	38 (4.5)	14 (15.2)		8.51(3.53–20.54)
Patient origin					
Endemic^d^	547 (58.4)	511 (60.5)	36 (39.1)	<.001	1 (ref)
Non/low endemic^e^	388 (41.4)	332 (39.3)	56 (60.9)		2.39 (1.54–3.72)
Missing	2 (0.2)	2 (0.2)	0		
Duration of residency, in patients with endemic origin					
<15 y	385 (70.4)	363 (71.0)	22 (61.1)	.11	1 (ref)
≥15 y	121 (22.1)	109 (21.3)	12 (33.3)		1.82 (.87–3.79)
Missing	41 (7.5)	39 (7.6)	2 (5.6)		
Sex					
Male	623 (66.5)	567 (67.1)	56 (60.9)	.23	1 (ref)
Female	314 (33.5)	278 (32.9)	36 (39.1)		1.31 (.84–2.04)
Region of infection^f^					
Eastern Africa	337 (36.0)	304 (36.0)	33 (35.9)		
Middle Africa	73 (7.8)	67 (7.9)	6 (6.5)		
Northern Africa	14 (1.5)	13 (1.5)	1 (1.1)		
Southern Africa	14 (1.5)	12 (1.4)	2 (2.2)		
Western Africa	452 (48.2)	409 (48.4)	43 (46.7)		
Africa, region unknown	2 (0.2)	2 (0.2)	0 (0)		
Americas^g^	5 (5.3)	5 (0.6)	0 (0)		
South Asia	8 (0.9)	5 (0.6)	3 (3.3)		
Southeast Asia	30 (3.2)	26 (3.1)	4 (4.4)		
Oceania	1 (0.11)	1 (0.12)	0 (0)		
Chemoprophylaxis use					
Regular	171 (18.3)	153 (18.1)	18 (19.6)	.70	1 (ref)
Irregular	136 (14.5)	125 (14.8)	11 (12.0)		0.75 (.34–1.64)
None	586 (62.5)	525 (62.1)	61 (66.3)		0.99 (.57–1.72)
Missing	44 (4.7)	42 (5.0)	2 (2.2)		
Patient delay, d					
Mean (SD)	3.69 (3.69)	3.67 (3.72)	3.73 (3.41)	.42	
0–1	221 (23.6)	200 (23.7)	21 (22.8)	.76	1 (ref)
2–3	335 (35.7)	303 (35.9)	31 (33.7)		0.97 (.54–1.74)
≥4	359 (38.31)	320 (37.9)	39 (42.4)		1.16 (.66–2.03)
Missing	23 (2.5)	22 (2.6)	1 (1.1)		
Health care delay, d					
Mean (SD)	0.7 (3.4)	0.6 (3.5)	1.1 (2.12)	.001	
0	734 (78.3)	678 (80.2)	56 (60.9)	.001	1 (ref)
1	66 (7.0)	53 (6.3)	13 (14.1)		2.97 (1.53–5.77)
≥2	83 (8.9)	63 (7.5)	20 (21.7)		4.74 (2.09–10.73)
Missing	54 (5.8)	51 (6.0)	3 (3.3)		
Pregnancy					
No. (%) of women	14 (4.5)	11 (4.0)	3 (8.3)	.21	2.21 (.59–8.32)

Abbreviations: CI, confidence interval; OR, odds ratio; SD, standard deviation.

^a^According to World Health Organization criteria for severe malaria [14] and/or hyperparasitemia >5% (Table 1).

^b^ORs estimated using univariable logistic regression.

^c^ *P* values estimated with χ^2^ test for categorical data and Wilcoxon-Mann-Whitney test for continuous data.

^d^Origin in countries of sub-Saharan Africa.

^e^Three hundred fifty-five from Sweden, 12 from other countries in Europe, 10 from Asia, 3 from South America, 4 from North America, 3 from Australia and New Zealand.

^f^Countries classified into regions according to United Nations geoscheme.

^g^South America and the Caribbean.

Ninety-two patients (9.8%) fulfilled the severe malaria definition, and 68 (7.3%) had severe criteria without hyperparasitemia as single criterion. One fatal outcome was reported in a Swedish man aged 43, corresponding to a case fatality ratio of 0.1%.

Healthcare delay, age, and non/low endemic origin were associated with severe malaria and included in the multivariable model. The odds of severe malaria increased with age (odds ratio [OR], from 1.73 [95% confidence interval {CI}, .80–3.74] in 30- to 39-year-olds to 8.51 [95% CI, 3.53–20.54] for age ≥60 years). Chemoprophylaxis usage did not differ significantly between severe and nonsevere cases (Table 2).

### Chronic Diseases and Severe Malaria

One hundred seventy-nine of 937 (19.1%) patients had at least 1 chronic condition; in multivariable analysis, having ≥2 diseases was associated with severe malaria (adjusted OR [aOR], 2.49 [95% CI, 1.10–5.68]), as was a Charlson score ≥1 (aOR, 2.63 [95% CI, 1.45–4.77]) (Table 3), with no effect modification by age (*P* = .46).

**Table 3.** Comorbidity in Patients with *Plasmodium falciparum* Malaria and Association With Severe Malaria, Defined According to World Health Organization Severe Criteria With and Without Hyperparasitemia as Single Criterion

Comorbidity	No. (%) of Patients		*P* Value^b^	OR (95% CI)		No. (%) of Patients		*P* Value^b^	OR (95% CI)	
	Nonsevere Malaria (n = 845)	Severe Malaria^a^ (n = 92)		Unadjusted^c^	Adjusted^d^	Nonsevere Malaria (n = 868)	Severe Malaria^e^ (n = 68)		Unadjusted^c^	Adjusted^d^
Chronic diseases										
Previously healthy	654 (77.4)	61 (66.3)	<.001	1 (ref)	1 (ref)	670 (77.2)	45 (66.2)	.01	1 (ref)	1 (ref)
1 chronic disease	122 (14.4)	17 (18.5)		1.49 (.84–2.65)	0.97 (.51–1.85)	124 (14.3)	15 (22.1)		1.8 (.97–3.33)	1.20 (.61–2.40)
≥2 chronic diseases	29 (3.4)	11 (12.0)		4.07 (1.94–8.54)	2.49 (1.10–5.68)	33 (3.8)	7 (10.3)		3.16 (1.32–7.54)	1.94 (.75–5.02)
Missing	40 (4.7)	3 (3.3)				41 (4.7)	1 (1.5)			
Charlson score										
≥1	84 (9.9)	22 (23.9)	<.001	2.82 (1.66–4.80)	2.63 (1.45–4.77)	89 (10.3)	17 (25.0)	<.001	2.82 (1.56–5.10)	2.59 (1.34–5.01)
Specific diagnosis^f^										
Asthma or COPD^g^	13 (1.5)	1 (1.1)	>.99	0.69 (.09–5.36)		13 (1.5)	1 (1.5)	>.99	0.95 (.12–7.36)	
Autoimmune diseases^h^	6 (0.7)	2 (2.2)	.19	3.06 (.61–15.4)	1.56 (.29–8.39)	6 (0.7)	2 (2.9)	.12	4.21 (.83–21.3)	1.77 (1.03–3.05)
Cardiovascular diseases^i^	17 (2.0)	7 (7.6)	.001	3.96 (1.60–9.82)	1.63 (.55–4.86)	20 (2.3)	4 (5.9)	.10	2.56 (.85–7.72)	0.85 (.22–3.33)
Chronic hepatitis^j^	23 (2.7)	1 (1.1)	.50	0.39 (.05–2.90)		23 (2.7)	1 (1.5)	>.99	0.53 (.07–3.98)	
Diabetes mellitus^k^	24 (2.8)	9 (9.8)	.001	3.66 (1.65–8.15)	2.98 (1.25–7.09)	25 (2.9)	8 (11.8)	<.001	4.35 (1.88–10.1)	3.71 (1.49–9.20)
HIV^l^	16 (1.9)	5 (5.4)	.03	3.00 (1.07–8.40)	5.37 (1.71–16.86)	18 (2.1)	3 (4.4)	.21	2.18 (.63–7.60)	3.42 (.89–13.12)
Hypertension^m^	35 (4.1)	13 (14.1)	<.001	3.76 (1.91–7.42)	1.57 (.71–3.46)	38 (4.4)	10 (14.7)	<.001	3.64 (1.73–7.69)	1.50 (.63–3.58)
Malignancies^n^	5 (0.6)	1 (1.1)	.47	1.82 (.21–15.7)		5 (0.6)	1 (1.5)	.37	2.49 (.29–21.6)	
Psychiatric disorders^o^	14 (1.7)	1 (1.1)	>.99	0.64 (.08–4.94)		15 (1.7)	0 (0)	.62		
Thyroid dysfunction^p^	11 (1.3)	1 (1.1)	>.99	0.82 (.10–6.43)		12 (1.4)	0 (0)	>.99		

Abbreviations: CI, confidence interval; COPD, chronic obstructive pulmonary disease; HIV, human immunodeficiency virus; OR, odds ratio.

^a^Defined according to World Health Organization (WHO) criteria for severe malaria [14] and/or hyperparasitemia >5% (Table 1).

^b^ *P* values estimated with χ^2^ test for all comparisons with cell counts >5 and with Fisher exact test for cell counts ≤5.

^c^Eight hundred ninety-four patients with information on comorbidity included in unadjusted analysis. Unadjusted ORs estimated with univariable logistic regression.

^d^Eight hundred forty-four patients with information on comorbidity and adjusted variables included. ORs adjusted for age, healthcare delay, and patient origin estimated with multivariable logistic regression. For cardiovascular disease and hypertension, additional adjustment for diabetes was made.

^e^Defined according to WHO criteria for severe malaria [14] without hyperparasitemia as single criterion. One individual without information on severe signs but known hyperparasitemia >5% excluded in analysis.

^f^Includes diagnosis present in at least 5 patients. Absence of diagnosis was used as reference for respective diagnosis in unadjusted and adjusted analyses.

^g^Includes all diagnoses of asthma (J45) and COPD (J44); all codes shown are from the *International Classification of Diseases, Tenth Revision* (*ICD-10*).

^h^Includes inflammatory systemic diseases (M30–M36), spondylopathies (M45–M49), and noninfectious inflammatory bowel diseases (K50–K52). None of the patients had ongoing steroid or immunosuppressive treatment reported in the medical record.

^i^Includes ischemic heart diseases (I20–I25), other heart diseases such as arrhythmias, cardiomyopathy, and heart failure (I30–I52), diseases of the cerebral arteries (I60–I69), medical conditions in the pulmonary circulation (I26–I28), or peripheral vascular disease (I70–I79, I80–I89).

^j^Chronic hepatitis B or C (B18.0–B18.2), confirmed microbiologically.

^k^Includes diabetes mellitus type 1 (E10) (4 patients), type 2 (E11) (23 patients), and unspecified (E14) (6 patients).

^l^HIV-infected patients were compared with patients without known HIV (264 tested negative and 652 untested). Fourteen patients with missing information on whether HIV test had been taken not included in analysis.

^m^Includes high blood pressure and related diseases (I10–I15).

^n^Malignant tumors (C00–C97). All of the malignancies had been previously operated or treated; none of the patients had a known active cancer or ongoing treatment.

^o^Mental diseases and syndromes and behavioral disorders (F00–F99).

^p^Includes all diseases of the thyroid gland (E00–E07).

Individual diagnoses associated with severe malaria in the univariable analysis were diabetes, hypertension, cardiovascular disease, and HIV (Table 3). After adjustment for age, healthcare delay, and patient origin, the associations remained significant for diabetes (aOR, 2.98 [95% CI, 1.25–7.09]) and HIV (aOR, 5.37 [95% CI, 1.71–16.86]). There was no overlap between HIV and diabetes; moreover, HIV resulted in a general drift in ORs, likely explained by small sample bias or noncollapsibility, thus not included in the final model.

Additional adjustment for hypertension and cardiovascular disease did not affect the association between diabetes and severity (OR, 2.75 [95% CI, 1.13–6.70]). Four of 33 diabetic patients had type 1 diabetes and the only type 1 diabetic patient with severe malaria had BMI 32.7, hypertension, and hyperlipidemia.

Hyperparasitemia >5% tended to be more frequent among diabetic compared with nondiabetic patients (15% [5/33] vs 7% [57/861]; *P* = .08). Nonetheless, among severe cases with diabetes, 4 of 9 had severe criteria without hyperparasitemia. Common clinical presentations of severe malaria among diabetics were respiratory distress with pulmonary edema, macroscopic hematuria, and renal impairment (Supplementary Table 1). A sensitivity analysis excluding hyperparasitemia >5% as single criterion resulted in an even stronger association between diabetes and severe malaria (aOR, 3.71 [95% CI, 1.49–9.20]), but not a significant association for HIV (Table 3).

The only detected interaction was between healthcare delay and diabetes, but as the interaction was weak (*P* = .04) and based on only 6 patients, it was not included in the multivariable model.

### BMI and Severe Malaria

Data on BMI were available for 219 of the 569 patients from Stockholm and Umeå. Patients with obesity were older, more often of endemic origin, and had more comorbidities (especially diabetes and hypertension), but were similar to nonobese patients regarding sex, chemoprophylaxis, patient and healthcare delay, and none with HIV (Supplementary Table 2).

Median BMI was higher among severe (29.3 [range, 18.45–54.1]) than nonsevere cases (24.7 [range, 18.45–47.1]) (*P* < .001), and 21.8% (12 of 55) of severe cases were obese (BMI >30) compared with 4.5% (23 of 514) of uncomplicated cases (*P* < .001). Obesity was highly associated with severity after adjusting for age, healthcare delay, and patient origin (aOR, 5.58 [95% CI, 2.03–15.36]), and also after additional adjustments for hypertension, diabetes, and cardiovascular disease (aOR, 4.32 [95% CI, 1.39–13.42]). The multiple imputed analysis yielded similar results as the complete case analysis (Table 4). No significant interactions were found between obesity and other variables in the multivariable model.

**Table 4.** Body Mass Index, Obesity, and Metabolic Risk Factors in Patients With *Plasmodium falciparum* Malaria From Stockholm and Umeå and Association With Severe Malaria, Defined According to World Health Organization Severe Criteria With and Without Hyperparasitemia as Single Criterion

Factor	No. (%) of Patients			*P* Value^b^	OR (95% CI)					
	Total (n = 569)	Nonsevere Malaria (n = 515)	Severe Malaria^a^ (n = 55)		Complete Case Analysis^a^ (n = 219)		Sensitivity Analysis^c^ (n = 219)		Imputed Analysis^d^ (n = 569)	
					Unadjusted^e^	Adjusted^f^	Unadjusted^e^	Adjusted^f^	Unadjusted^g^	Adjusted^h^
BMI, kg/m^2^										
18.5–24.9	117 (20.6)	107 (20.8)	10 (18.2)	<.001	1 (ref)	1 (ref)	1 (ref)	1 (ref)		
25–29.9	67 (11.8)	62 (12.1)	5 (9.1)		0.86 (.28–2.64)	0.84 (.25–2.84)	1.42 (.37–5.49)	1.72 (.38–7.78)		
≥30	35 (6.2)	23 (4.5)	12 (21.8)		5.58 (2.15–14.47)	5.18 (1.67–16.02)	10.27 (3.27–32.27)	11.20 (2.73–46.00)		
Missing	350 (61.5)	322 (62.7)	28 (50.9)							
Obesity^i^	35 (6.2)	23 (4.5)	12 (21.8)	<.001	5.88 (2.45–14.10)	5.58 (2.03–15.36)	8.91 (3.35–23.72)	8.63 (2.70–27.63)	5.14 (2.10–12.51)	4.39 (1.66–11.58)
Metabolic risk factors^j^	68 (12.0)	49 (9.5)	19 (34.6)	<.001	5.50 (2.45–12.35)	4.05 (1.57–10.48)	8.94 (3.35–23.89)	6.62 (2.08–21.06)	5.56 (2.53–12.21)	4.06 (1.81–9.11)
Metabolic syndrome^k^	14 (2.5)	7 (1.4)	7 (12.7)	<.001	9.25 (2.94–29.06)	6.54 (1.87–22.88)	10.23 (3.11–33.62)	7.01 (1.85–26.52)	7.15 (2.66–19.18)	4.29 (1.35–13.68)

Abbreviations: BMI, body mass index; CI, confidence interval; OR, odds ratio.

^a^Severe malaria defined according to World Health Organization (WHO) criteria for severe malaria [14] and/or hyperparasitemia >5% (Table 1).

^b^ *P* values estimated with χ^2^ test for categorical data and Wilcoxon-Mann-Whitney test for continuous data.

^c^Severe malaria defined according to WHO criteria [14] without hyperparasitemia as single criterion.

^d^Missing obesity status imputed using multiple imputation by chained equations including age, weight, severe malaria, treatment in intensive care unit, year of diagnosis, healthcare delay, HIV status, sex, endemicity of patient origin, and comorbidity (cardiovascular disease, diabetes, hypertension).

^e^Unadjusted ORs estimated with univariable logistic regression for patients with information on BMI.

^f^ORs adjusted for age, healthcare delay, and patient origin estimated with multivariable logistic regression for patients with information on BMI.

^g^Imputed output used in univariable logistic regression to estimate unadjusted ORs.

^h^Imputed output used in multivariable logistic regression to estimate ORs adjusted for age, healthcare delay, and patient origin.

^i^Obesity defined as BMI ≥30 kg/m^2^ (obesity class I–III according to WHO BMI classification [19]).

^j^Patients with at least 1 metabolic risk factor (obesity, hypertension, diabetes, or dyslipidemia).

^k^Adjusted version of International Diabetes Federation’s criteria [20] for metabolic syndrome: Obesity (BMI ≥30 kg/m^2^) together with at least 1 additional risk factor (hypertension, diabetes, or dyslipidemia).

Hyperparasitemia >5% was more common among obese (17.1% [6/35]) than nonobese (4.9% [9/184]; *P* = .009) patients, mainly reflecting parasitemias ≥10% (11.4% [4/35] vs 2.7% [5/184]; *P* = .02); however, among severe cases with obesity, 6 of 12 had severe criteria without hyperparasitemia (Table 5).Common severe presentations in obese patients were respiratory distress and circulatory collapse (Supplementary Table 1). As for diabetes, the association between obesity and severe malaria became stronger when hyperparasitemia was excluded as a single criterion (aOR, 8.63 [95% CI, 2.70–27.63]) (Table 4).

**Table 5.** *Plasmodium falciparum* Malaria Parasitemia and Severity in Relation to Diabetes and Obesity

Parasitemia Status	No. (%) of Patients in Whole Study Population				No. (%) of Patients in Population With BMI Assessment			
	Total^a^ (n = 937)	Nondiabetics (n = 861)	Diabetics (n = 33)	*P* Value^b^	Total^c^ (n = 569)	Nonobese (n = 184)	Obese (n = 35)	*P* Value^b^
Parasitemia^d^								
≤0.1	238 (25.4)	220 (25.6)	10 (30.3)	.08	166 (29.2)	38 (20.7)	11 (31.4)	.04
0.2–1.0	298 (31.8)	284 (33.0)	5 (15.2)		207 (36.4)	78 (42.4)	9 (25.7)	
1.1–2.0	134 (14.3)	120 (13.9)	6 (18.2)		85 (14.9)	28 (15.2)	6 (17.1)	
2.1–5	107 (11.4)	97 (11.3)	5 (15.1)		65 (11.4)	28 (15.2)	3 (8.6)	
5.1–10	35 (3.7)	31 (3.6)	2 (6.1)		21 (3.7)	4 (2.2)	2 (5.7)	
>10	30 (3.2)	26 (3.0)	3 (9.1)		17 (3.0)	5 (2.7)	4 (11.4)	
Missing	95 (10.1)	83 (9.6)	2 (6.1)		8 (1.4)	3 (1.6)	0 (0)	
Hyperparasitemia								
>5%	90 (9.6)	57 (6.6)	5 (15.1)	.08	38 (6.7)	9 (4.9)	6 (17.1)	.01
Severity								
Nonsevere	845 (90.2)	781 (90.7)	24 (72.7)	.004	514 (90.3)	169 (91.9)	23 (65.7)	<.001
Hyperparasitemia >5% only	23 (2.5)	21 (2.4)	1 (3.0)		13 (2.3)	6 (3.3)	1 (2.9)	
Severe criteria only^e^	27 (2.9)	23 (2.7)	4 (12.1)		17 (3.0)	6 (3.3)	6 (17.1)	
Severe criteria and hyperparasitemia	41 (4.4)	36 (4.2)	4 (12.1)		24 (4.2)	3 (1.6)	5 (14.3)	
Hyperparasitemia, unknown severe signs	1 (0.1)	0	0		1 (0.2)	0	0	

Abbreviation: BMI, body mass index.

^a^Forty-three patients had missing data for diabetes status.

^b^ *P* values estimated with Fisher exact test.

^c^Three hundred fifty patients had missing data for BMI.

^d^Maximum parasitemia observed during malaria episode.

^e^Clinical, radiological, or laboratory findings of severe malaria as defined according to World Health Organization [14] (Table 1). For 2 patients with severe criteria, the parasitemia was unknown.

Having at least 1 metabolic risk factor (hypertension, dyslipidemia, diabetes, and/or obesity) was associated with severe malaria (aOR, 4.05 [95% CI, 1.57–10.48]), as was obesity combined with ≥1 additional metabolic risk factor, corresponding to 2 of 3 criteria for metabolic syndrome [20] (aOR, 6.54 [95% CI, 1.87–22.88]) (Table 4). Only 5 of 219 patients fulfilled the complete metabolic syndrome criteria (3 with severe malaria), thus were too few to analyze. In the subset with collected BMI data, diabetes was associated with severe malaria (aOR, 3.37 [95% CI, 1.19–9.54]), and adjusting for obesity reduced the aOR to 2.39 (95% CI, .61–9.32).

### Patient Origin and Time in Nonendemic Country

Obesity and diabetes were most prevalent among patients of endemic origin with long residency in Sweden: 16.5% (15/91), 4.8% (12/248), and 3.8% (8/209) for obesity (*P* = .001), and 9.1% (11/121), 2.3% (9/385), and 3.4% (13/388) (*P* = .004) for diabetes in patients with residency ≥15 years, <15 years, and non/low-endemic origin, respectively. Stratified analyses resulted in positive associations between both obesity and diabetes and severe malaria in all categories; however, only significantly among patients of endemic origin with residency ≥15 years (obesity: aOR, 6.88 [95% CI, 1.21–39.24]; diabetes: aOR, 5.32 [95% CI, 1.01–28.18]). Interaction analysis could not verify that patient origin, and time of residency modified the association of either diabetes or obesity with severity (all *P* > .50).

## DISCUSSION

In this nationwide study including 937 adults with *P. falciparum* malaria in Sweden, we identified comorbidity, and specifically diabetes, obesity, and components of the metabolic syndrome, as risk factors for severe malaria both in nonimmune travelers and immigrants from sub-Saharan Africa.

Patients with ≥2 chronic diseases or a Charlson score ≥1 had an increased risk of severe malaria. Moreover, age, healthcare delay, nonendemic origin, and HIV were associated with severe malaria as previously shown [4, 8, 11]. However, few patients were HIV infected, and the association with severity became nonsignificant when excluding hyperparasitemia as single criterion for severe malaria.

Diabetes, hypertension, and cardiovascular disease were all associated with severe malaria in the univariable analysis; however, diabetes was the only NCD that on its own remained significant after adjustments. Only a few had type 1 diabetes, so conclusions can only be drawn for type 2 diabetes. Other NCDs might also affect the outcome of malaria; however, prevalence in these travelers was too low to assess their impact.

Obesity was highly associated with severity in the population subset with retrieved BMI data. Type 2 diabetes is a complication of obesity and the 2 metabolic disorders often coexist. Adjusting for obesity somewhat reduced the odds of severe malaria among diabetics, whereas adjusting for the potential intermediate factors diabetes, hypertension, and cardiovascular disease [23] did not substantially change the association between obesity and severity. Having at least 1 metabolic risk factor was associated with increased odds for severe malaria, and a combination including obesity conferred even higher odds.

Reports on NCDs and malaria are few. To our knowledge, no study has assessed the association between diabetes and severe malaria. Two reports from Ghana have shown that semi-immune adults with type 2 diabetes were more susceptible to *Plasmodium* infection than controls [7, 24], but with no inference on severity. A recent animal study indicates enhanced transmission of parasites from diabetics to mosquitoes [25].

BMI in relation to malaria has mainly been assessed in populations with considerably lower BMI [6, 26, 27]. Underweight was identified as a protective factor against severe malaria [26],whereas overweight was associated with progression to severe malaria after treatment start [6], and fatal cases had higher BMI (mean, 25.3) compared with nonfatal severe cases (20.4) [28]. However, none of these studies investigated the effect of obesity. Here, median BMI was higher among severe cases (29.3) than nonsevere cases (24.7); and obesity (BMI ≥30), but not overweight (BMI 25–29), was strongly associated with severe malaria at diagnosis.

Obesity and diabetes (type 1 and 2) are well recognized to increase severity of infections [29, 30]. Both conditions are characterized by low-grade chronic inflammation and altered levels of nutrients and metabolic hormones with immunomodulatory effects [30, 31]. Equally important, metabolic changes could have specific effects on the malaria parasite and pathogenesis. Acute malaria is well recognized to influence plasma glucose and lipid levels [14, 32, 33]. Parasite growth in vitro is affected by glucose levels [34]; in Ghana, diabetic adults had 5% greater risk of asymptomatic parasitemia for each millimolar increase of plasma glucose [7]. Moreover, lipoproteins are important for parasite cell membranes and endothelial adherence of infected erythrocytes [35, 36].

Obese patients had higher parasitemia compared with nonobese patients (especially >10%), a feature also described in obese mice [37], and hyperparasitemia >5% tended to be more common in diabetics. Nonetheless, approximately half of diabetic patients and obese patients with severe malaria had severe criteria without hyperparasitemia, and the associations with severity were even stronger when hyperparasitemia was excluded as single severe criterion, suggesting that parasitemia could not solely explain the more severe presentations. The mechanisms by which these comorbidities affect malaria pathology clearly need to be further investigated.

Numerous studies have shown that immigrants from high-endemic regions are at lower risk of severe and fatal malaria compared to nonimmune travelers [9–11]. Recently, we found that immigrants from sub-Saharan Africa residing ≥15 years in Sweden had a similar risk of severe malaria as nonimmune travelers [22]. Interestingly, both obesity and diabetes were most prevalent in this group. Poor antibody response after vaccination toward bacterial and viral infections has been observed in obese individuals [31]; hence, lifestyle diseases such as diabetes and obesity might possibly affect maintenance of immunity acquired against severe malaria. Although the association with severe malaria was strongest among patients of endemic origin with residency ≥15 years, larger studies are needed to truly assess possible interaction.

Our study has several limitations. The retrospective design could lead to misclassification of exposures and outcome variables. The current data were entered as part of a larger epidemiological study without priori hypotheses. Moreover, in Sweden, fever after tropical visits is managed by infectious diseases specialists using standardized protocols. A high sensitivity of comorbidity assessment was achieved by using medical records, registered *ICD* codes, and medication lists. Moreover, prevalence data on chronic diseases and BMI present before the malaria episode produce an acceptable proxy for longitudinal data. However, a majority of the HIV tests were taken in conjunction with the malaria episode and severe cases were more often tested, which could imply a detection bias for HIV. Nonetheless, 18 of 21 HIV cases were known before, and among the 3 newly detected, none had severe malaria. Data on BMI were lacking from a large proportion of patients. Incorporating the factors associated with missing data (Supplementary Table 2) in a multiple imputation model resulted in similar effect of obesity on severity as the complete case analysis. Systematic health screening including BMI and glucose control, preferably in prospective studies, are needed to confirm our results. Possibly, some patients had yet undiagnosed chronic diseases, such that nondifferential misclassification might have diluted the effects.

The strength of our study is that it includes clinical data from a large population of travelers, with a mixed constitution of individuals previously exposed and unexposed to malaria, diagnosed at multiple centers. Case detection was based on the national surveillance system with high detection sensitivity [13]. The results are well generalizable to settings with a similar spectrum of imported malaria.

We believe our findings have implications beyond the management of malaria in travelers. Sub-Saharan Africa is facing a double burden of disease; while continuing to deal with malaria and other infections, there is a rapid upsurge in NCDs [1]. This is especially alarming considering that an estimated two-thirds of diabetic individuals in the African region are undiagnosed [38]. Moreover, with the recent changes in malaria transmission in many areas, natural acquired immunity against uncomplicated and severe malaria will probably be affected [39]. Increasing prevalence of comorbidities such as obesity and diabetes might make these populations more vulnerable to severe malaria.

## CONCLUSIONS

This is, to our knowledge, the first study investigating the impact of NCDs on severity of *P. falciparum* malaria. We show that obesity, diabetes, and combinations of metabolic risk factors were associated with severe malaria, both in nonimmune travelers and immigrants from sub-Saharan Africa. The findings are of high clinical relevance for the acute management of malaria in travelers; there is also an urgent need for awareness and further investigations of these risk factors in malaria-endemic areas.

## Supplementary Data

Supplementary materials are available at *Clinical Infectious Diseases* online. Consisting of data provided by the authors to benefit the reader, the posted materials are not copyedited and are the sole responsibility of the authors, so questions or comments should be addressed to the corresponding author.

## Supplementary Material

Wyss_etal_CID_85276_Supplementary_Material_clean

## Notes

***Author contributions.*** Study concept and design: A. F., P. N., K. W. Data acquisition, analysis, or interpretation: all authors. Statistical analysis: U. F., K. W., P. N. Drafting the manuscript: K. W., A. F., M. V. Revising the manuscript critically for important intellectual content: all authors.

***Acknowledgments.*** We thank all Departments of Infectious Diseases for contributing with medical records; Therese Djärv and Victor Yman for methodological advice; and Marika Hjertquist and Elsie Ydring for contributing with data from the Public Health Agency database.

***Disclaimer.*** The funders had no role in the design and conduct of the study; the collection, analysis, and interpretation of the data; the preparation of the manuscript; or the decision to submit the manuscript for publication.

***Financial support.*** This work was supported by the Swedish Research Council (grant numbers 348-2013-6573 and 2015–09277) and the Stockholm County Council (grant numbers 20130207 and 20150135).

***Potential conflicts of interest.*** All authors: No potential conflicts of interest. All authors have submitted the ICMJE Form for Disclosure of Potential Conflicts of Interest. Conflicts that the editors consider relevant to the content of the manuscript have been disclosed.
