# Supplementary material for: Obesity and Diabetes as Risk Factors for Severe Plasmodium falciparum Malaria: Results From a Swedish Nationwide Study
Source: Clin Infect Dis. 2017 May 16;65(6):949–58. doi: 10.1093/cid/cix437 (PMC5848256; doi:10.1093/cid/cix437)
Supplement: Wyss_etal_CID_85276_Supplementary_Material_clean [file cix437_suppl_wyss_etal_cid_85276_supplementary_material_clean.docx]

**Supplementary Material**

**Supplementary Table 1. Clinical presentation of severe *Plasmodium falciparum* malaria in relation to diabetes and obesity**

**Supplementary Table 2. Characteristics of patients with *Plasmodium falciparum* malaria from Stockholm and Umeå according to obesity status and missingness of BMI data**

**Supplementary Table 1. Clinical presentation of severe *Plasmodium falciparum* malaria in relation to diabetes and obesity**

| **Severe criteria^a^** | **No. (%) of severe cases in whole study population** | | | **No. (%) of severe cases in Stockholm Umeå population** | | |
| --- | --- | --- | --- | --- | --- | --- |
|  | **Total^b^**  (n=92) | **Non-diabetics**  (n=80) | **Diabetics**  (n=9) | **Total^c^**  (n=55) | **Non-obese**  (n=15) | **Obese**  (n=12) |
| Unrousable coma | 19 (20.7) | 17 (21.3) | 2 (22.2) | 10 (18.2) | 2 (13.3) | 2 (16.7) |
| Multiple convulsions | 7 (7.6) | 6 (7.5) | 1 (11.1) | 2 (3.6) | 0 (0) | 0 (0) |
| Respiratory distress | 34 (37.0) | 29 (36.3) | 4 (44.4) | 18 (32.7) | 7 (46.7) | 5 (41.7) |
| Circulatory collapse | 23 (25.0) | 20 (25.0) | 2 (22.2) | 14 (25.4) | 5 (33.3) | 5 (41.7) |
| Pulmonary oedema | 13(14.1) | 9 (11.3) | 4 (44.4) | 8 (14.6) | 4 (26.7) | 4 (33.3) |
| ARDS | 13 (14.1) | 11 (13.8) | 2 (22.2) | 5 (9.1) | 2 (13.3) | 1 (8.3) |
| Renal impairment | 21 (22.8) | 17 (21.3) | 3 (33.3) | 10 (18.2) | 2 (13.3) | 3 (25.0) |
| Acidosis | 13 (14.1) | 10 (12.5) | 2 (22.2) | 8 (14.6) | 1 (6.7) | 3 (25.0) |
| Jaundice | 36 (39.1) | 33 (41.3) | 2 (22.2) | 17 (30.9) | 5 (33.3) | 2 (16.7) |
| Severe anemia | 6 (6.5) | 6 (7.5) | 0 (0) | 3 (5.5) | 0 (0) | 1 (8.3) |
| Abnormal bleeding | 10 (10.9) | 10 (12.5) | 0 (0) | 6 (10.9) | 3 (20.0) | 1 (8.3) |
| Macroscopic hemoglobinuria | 23 (25.0) | 18 (22.5) | 4 (44.4) | 12 (21.8) | 0 (0) | 3 (25.0) |
| Hypoglycemia | 1 (1.1) | 1 (1.25) | 0 (0) | 1 (1.8) | 1 (6.7) | 0 (0) |

Abbreviations; ARDS, acute respiratory distress syndrome

^a^ Clinical, radiological or laboratory finding of severe malaria as defined according to WHO [14] (Table 1).

^b^ Of which 3 with missing data for diabetes status.

^c^ Of which 28 with missing data for BMI.

**Supplementary Table 2. Characteristics of patients with *Plasmodium falciparum* malaria from Stockholm and Umeå according to obesity status and missingness of BMI data**

|  | **No. (%) of patients according to obesity status (n=219)** | | | **No. (%) of patients according to missingness (n=569)** | | |
| --- | --- | --- | --- | --- | --- | --- |
|  | **Non-obese (BMI<30)**  n =184 | **Obese (BMI≥30)**  n =35 | **P value** | **Patients with data on weight and height** n=219 | **Patients with missing**  **BMI data^a^**  n= 350 | **P value** |
| **Age, y** |  |  |  |  |  |  |
| median (range) | 36 (19-83) | 48.1 (29-66) | <.001 | 38.5 (19-83) | 37 (18-67) | .10 |
| 18-29 | 53 (28.8) | 1 (2.9) |  | 54 (24.7) | 83 (23.7) | .007 |
| 30-39 | 56 (30.4) | 7 (20.0) |  | 63 (28.8) | 131 (37.4) |  |
| 40-49 | 34 (18.5) | 15 (42.9) |  | 49 (22.4) | 88 (25.1) |  |
| 50-59 | 25 (13.6) | 10 (28.6) |  | 35 (16.0) | 38 (10.9) |  |
| ≥ 60 | 16 (8.7) | 2 (5.7) |  | 18 (8.2) | 10 (2.9) |  |
| **Patient origin** |  |  |  |  |  |  |
| Endemic^b^ | 94 (51.1) | 27 (77.1) | .004 | 121 (55.3) | 239 (68.3) | .002 |
| **Patient origin and time in non-endemic country^c^** |  |  |  |  |  |  |
| Endemic <15 years | 58 (31.5) | 12 (34.3) | .001 | 70 (32.0) | 178 (50.9) | <.001 |
| Endemic ≥15 years | 31 (16.9) | 15 (42.9) |  | 46 (21.0) | 45 (12.9) |  |
| Non/low endemic | 90 (48.91) | 8 (22.9) |  | 98 (44.8) | 111 (31.7) |  |
| Unknown time of residency | 5 (2.72 ) | 0 (0) |  | 5 (2.3) | 16 (4.6) |  |
| **Sex** |  |  |  |  |  |  |
| Male | 130 (70.7) | 22 (62.9) | .36 | 152 (69.4) | 227 (64.9) | .26 |
| **Chemoprophylaxis use** |  |  |  |  |  |  |
| Regular | 32 (17.4) | 6 (17.1) | .92 | 38 (17.4) | 50 (14.3) | .77 |
| Irregular | 30 (16.3) | 4 (11.4) |  | 34 (15.5) | 56 (16.0) |  |
| No use | 111 (60.3) | 21 (60.0) |  | 132 (60.3) | 222 (63.4) |  |
| missing | 11 (6.0) | 4 (11.4) |  | 15 (6.9) | 22 (6.3) |  |
| **Patient delay, days** |  |  |  |  |  |  |
| mean (SD) | 3.71 (3.55) | 3.77 (2.91) | .52 | 3.72 (3.45) | 3.85 (3.98) | .96 |
| 0-1 | 39 (21.2) | 5 (14.3) | .48 | 44 (20.1) | 87 (24.9) | .29 |
| 2-3 | 71 (38.6) | 17 (48.6) |  | 88 (40.2) | 121 (34.6) |  |
| ≥ 4 | 73 (39.7) | 13 (37.1) |  | 86 (39.3) | 139 (39.7) |  |
| missing | 1 (0.5) | 0 (0) |  | 1 (0.5) | 3 (0.9) |  |
|  |  |  |  |  |  |  |
| **Supplementary Table 2. Characteristics of patients with *Plasmodium falciparum* malaria from Stockholm and Umeå according to obesity status and missingness of BMI data (continued)** | | | | | | |
|  | **Non-obese (BMI<30)** | **Obese (BMI≥30)** | **P value** | **Patients with data on weight and height** | **Patients with missing**  **BMI data^a^** | **P value** |
| **Health care delay, days** |  |  |  |  |  |  |
| mean (SD) | 0.45 (.62) | 0.8 (2.31) | .21 | 0.51 (1.75) | 0.4 (1.46) | .80 |
| 0 | 149 (80.98) | 27 (77.14) | .29 | 176 (80.4) | 264 (75.4) | .56 |
| 1 | 10 (5.4) | 4 (11.4) |  | 14 (6.4) | 29 (8.3) |  |
| ≥2 | 15 (8.2) | 4 (11.4) |  | 19 (8.7) | 25 (7.1) |  |
| missing | 10 (5.43) | 0 (0) |  | 10 (4.6) | 32 (9.1) |  |
| **Chronic diseases** |  |  |  |  |  |  |
| Previously healthy | 132 (71.7) | 19 (54.3) | .02 | 151 (69.0) | 296 (84.3) | <.001 |
| 1 chronic disease | 38 (20.7) | 8 (22.9) |  | 46 (21.0) | 42 (12.0) |  |
| ≥2 chronic diseases | 14 (7.6) | 8 (22.9) |  | 22 (10.1) | 9 (2.9) |  |
| missing | 0 (0) | 0 (0) |  | 0 | 3 (0.9) |  |
| **Specific diagnosis** |  |  |  |  |  |  |
| Cardiovascular disease^d^ | 10 (5.4) | 3 (8.6) | .44 | 13 (5.9) | 9 (2.6) | .045 |
| Diabetes mellitus^e^ | 3 (1.6) | 10 (28.6) | <.001 | 13 (5.9) | 7 (2.0) | .01 |
| Hypertension^f^ | 13 (7.1) | 8 (22.9) | .004 | 21 (9.6) | 11 (3.1) | .001 |
| Dyslipidemia^g^ | 2 (1.1) | 2 (5.7) | .12 | 4 (1.8) | 0 | .02 |
| HIV | 6 (3.3) | 0 (0) | .59 | 6 (2.7) | 6 (1.7) | .43 |
| **Year of diagnosis** |  |  |  |  |  |  |
| 1995-1999 | 47 (25.5) | 5 (14.3) | .26 | 52 (23.7) | 122 (34.9) | <.001 |
| 2000-2004 | 29 (15.8) | 7 (20.0) |  | 36 (16.4) | 116 (33.1) |  |
| 2005-2009 | 44 (23.9) | 6 (17.1) |  | 50 (22.8) | 54 (15.4) |  |
| 2010-2015 | 64 (34.8) | 17 (48.6) |  | 81 (37.0) | 58 (16.6) |  |
| **Disease severity** |  |  |  |  |  |  |
| Severe malaria^h^ | 15 (8.2) | 12 (34.3) | <.001 | 27 (12.3) | 28 (8.0) | .09 |
| **Level of health care** |  |  |  |  |  |  |
| Intensive care unit | 13 (7.1) | 5 (14.3) | .18 | 18 (8.2) | 22 (6.3) | .38 |

Abbreviations: OR, odds ratio; CI, confidence interval; SD, standard deviation; BMI, body mass index

^a^ For 33 patients with missing BMI data, weight was known but not height, this data was used in the imputation.

^b^ Origin in countries of Sub-Saharan Africa.

^c^ Patients with origin in endemic countries of Sub-Saharan Africa with residency <15 years in Sweden compared to patients with endemic origin and residency ≥15 years and patients with origin in non or low endemic countries.

^d^ Includes ischaemic heart diseases (I20-I25), other heart diseases such as arrhythmias, cardiomyopathy and heart failure (I30-I52), diseases of the cerebral arteries (I60-I69), medical conditions in the pulmonary circulation (I26-I28) or peripheral vascular disease (I70-I79, I80- I89).

^e^ Includes diabetes mellitus type 1 (E10), type 2 (E11) and unspecified (E14).

^f^ Includes high blood pressure and related diseases (I10-I15).

^g^ Includes known diagnosis of hyperlipidaemia (E78).

^h^ According to WHO criteria for severe malaria [14] and/or hyperparasitemia >5%.
